# Factoring attitudes towards armed conflict risk into selection of protected areas for conservation

**DOI:** 10.1038/ncomms11042

**Published:** 2016-03-30

**Authors:** E. Hammill, A. I. T. Tulloch, H. P. Possingham, N. Strange, K. A. Wilson

**Affiliations:** 1 School of Biological Sciences, The University of Queensland, Brisbane, Queensland 4072, Australia; 2 Department of Watershed Sciences and the Ecology Center, Utah State University, Logan, Utah 84341, USA; 3 Department of Life Sciences, Imperial College London, Silwood Park, Berkshire SL5 7QN, UK; 4 Department of Food and Resource Economics, Centre for Macroecology, Evolution and Climate Change, University of Copenhagen, Rolighedsvej 23, Frederiksberg C DK-1958, Denmark

## Abstract

The high incidence of armed conflicts in biodiverse regions poses significant challenges in achieving international conservation targets. Because attitudes towards risk vary, we assessed different strategies for protected area planning that reflected alternative attitudes towards the risk of armed conflicts. We find that ignoring conflict risk will deliver the lowest return on investment. Opting to completely avoid conflict-prone areas offers limited improvements and could lead to species receiving no protection. Accounting for conflict by protecting additional areas to offset the impacts of armed conflicts would not only increase the return on investment (an effect that is enhanced when high-risk areas are excluded) but also increase upfront conservation costs. Our results also demonstrate that fine-scale estimations of conflict risk could enhance the cost-effectiveness of investments. We conclude that achieving biodiversity targets in volatile regions will require greater initial investment and benefit from fine-resolution estimates of conflict risk.

## Abstract (web summary)

Armed conflicts pose significant problems for ensuring that regional conservation plans are implemented. Here, Hammill *et al*. show that offsetting the impacts of protected area loss through protection of additional areas during conflicts provides the greatest return on investment for conservation programs.

## Graphical abstract

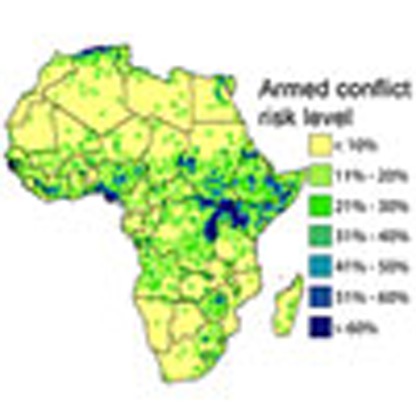

The establishment of effective protected areas remains at the heart of global conservation efforts^1^,^2^. Many terrestrial ecosystems are now so modified by humans that protected areas represent final refuges for threatened species^3^. However, the effectiveness of protected areas may be undermined when managers have insufficient resources to mitigate threats such as illegal logging^4^ and poaching^5^, alterations to environmental conditions^6^ and armed conflict^7^,^8^. The threat of armed conflict is of particular concern given the high occurrence of conflict in biodiversity hotspots^9^. Impacts of conflict include harvesting of valuable animal parts to fund paramilitaries^10^, reliance on fauna and flora for subsistence^11^, and collateral damage from military operations^12^. In addition to impacting biodiversity, conflict may also damage park infrastructure and imperil staff^13^,^14^,^15^. Despite evidence of conflict-related damage to species and protected areas, the effect of conflict remains complex, and in some cases could even benefit biodiversity by creating exclusion zones^16^ or hindering extractive industries^17^.

At a minimum, systematic conservation planning combines data on conservation features (for example, species' ranges) and management costs to identify areas for protection^18^,^19^. Typically, it is assumed that once designated, protected areas will mitigate threats to biodiversity. As conflict has the potential to undermine protected areas^20^, factoring conflict risk (a combination of the probability of a conflict occurring and its impact on protected areas) into the initial planning stages is crucial to ensure that conservation funds are optimally allocated. Conservation managers may face severe consequences if projects fail, and this fear of failure may lead to suboptimal, overly risk-averse management practices^21^,^22^. It is therefore necessary to quantify the conservation outcomes of different attitudes towards risk, so managers may tailor their practices to improve management outcomes and justify their actions.

Initial attempts at systematic conservation planning utilized coarse national-scale cost and biodiversity data^23^. The use of national-scale data is inefficient, as biodiversity and conservation costs are not uniformly distributed within nations. Improvements in data collection made fine-scale conservation planning possible^24^,^25^, yet conflict risk still tends to be reported at a national scale^26^,^27^,^28^. However, most conflicts take place at the intra-national level^28^, meaning localized areas experience various levels of conflict risk (often elevated along international borders^29^,^30^). Fine-scale estimates of conflict risk would have the benefit of accounting for within-nation spatial variability in conflict, thereby increasing the spatial precision of conservation planning.

To understand how conflict has affected conservation spending historically, we correlated domestic spending and international aid^31^ with national estimates of conflict risk^26^ and national-scale biodiversity^31^ (calculated by summing the fraction of the range for each species range within a nation^31^). In addition, we investigated correlations between biodiversity and conflict risk^9^. We then investigated four strategies for future protected area investment that reflect different attitudes towards conflict risk. In the first strategy, conflict risk is ignored and investment decisions are based on biodiversity and cost data alone (henceforth termed ‘conflict-ignorant'). Under the second strategy, areas with conflict-risk estimates above a designated threshold are excluded from selection (henceforth termed ‘conflict-avoiding'). The third strategy explicitly accounts for conflict risk when selecting protected areas (henceforth termed ‘conflict-accounting'). Under this conflict-accounting strategy, high-risk areas are avoided when others are available, and in the absence of alternatives, additional areas are selected to increase the chance that sufficient areas are protected to meet the conservation targets^32^. For example, if a conservation feature existed in 10 planning units that have a 25% chance of being impacted by conflict, protecting six planning units would give a 96.2% of reaching a target of protecting 30% of its range (binomial distribution, chance of three or less failures from six binomial trials where the chance of failure in one trial is 25%). The final strategy (henceforth termed as ‘conflict-sensitive') combines conflict-avoiding and conflict-accounting. Areas with a conflict-risk level above a threshold are unavailable (as for conflict-avoiding), and conflict risk in the remaining areas is accounted for (as for conflict-accounting). For example, if the maximum-risk threshold were 35%, a location with a 30% risk of conflict would remain available for selection, but this risk would be accounted for in the manner described above.

We used Africa as a test case to estimate how incorporating conflict risk affects conservation outcomes. Africa currently has ∼2.2 million km^2^ (7%) of its landmass protected^33^ (Supplementary Fig. 1), which is substantially less than the global average (13%). Across all African mammals, the mean proportion of species' ranges in protected areas is 17%, and >20% of species have <5% of their ranges protected^34^. Inadequate protection compromises the survival of many iconic mammal species, highlighting a need to increase the extent and effectiveness of protected areas^35^,^36^. However, many African nations have low ‘Peace Scores'^26^, calculated using levels of domestic and international conflict, level of national discord and level of militarization^26^. Africa's low peace scores are influenced by its history of serious incidents including civil war (Democratic Republic of the Congo, DRC) and genocide (Rwanda), many of which have directly impacted conservation efforts. During the Rwandan genocide, for example, the Akagera National Park was reduced to 30% of its original size^37^. The impact of conflict not only reduces the size of protected areas but can also reduce their effectiveness at conserving species, illustrated by conflict increasing poaching in central Africa^17^. At the end of 2014, it was estimated that 24 armed conflicts were ongoing in Africa^38^.

We used 100 runs of the software package Marxan to select protected area networks and incorporated conflict risk using the four different strategies. A grid was superimposed over Africa, dividing the continent into 10 km^2^ planning units. The amount of each conservation feature within each planning unit was calculated along with costs of purchasing and maintenance. Each planning unit was also assigned a conflict risk using two different methods: national-scale estimates from published sources (presented in the text^26^, Supplementary Note 3 and ref. 39), and a planning unit-specific estimate predicted from local historical armed conflicts^30^ (see Methods section, Supplementary Note 1, Supplementary Table 1 and Supplementary Fig. 2 for details).

For each conservation feature, we set a target of including 30% of their existing distribution in a future protected area network (a rule-of-thumb recommendation for terrestrial species^40^). The conservation importance of each planning unit was determined by its selection frequency within the 100 Marxan runs. In addition, for each investment strategy, the run that met all conservation targets at the least cost (representing the ‘best' solution) was used in later analyses. To evaluate the performance of the protected area networks, we used Monte Carlo simulations to estimate which planning units in the protected area network would be impacted by conflict during a 5-year management period. We compare the performance of the protected area networks using the planning units deemed unaffected by conflict. To quantify performance, we reported the number of conservation targets met, the cost of the protected area network and the return on investment (in terms of conservation targets met per $billion expended). We show that the conflict-accounting and conflict-sensitive strategies are the best performing, and this performance is enhanced by fine-scale conflict-risk data.

## Results

### Historical patterns of conservation spending

**Figure 1:**
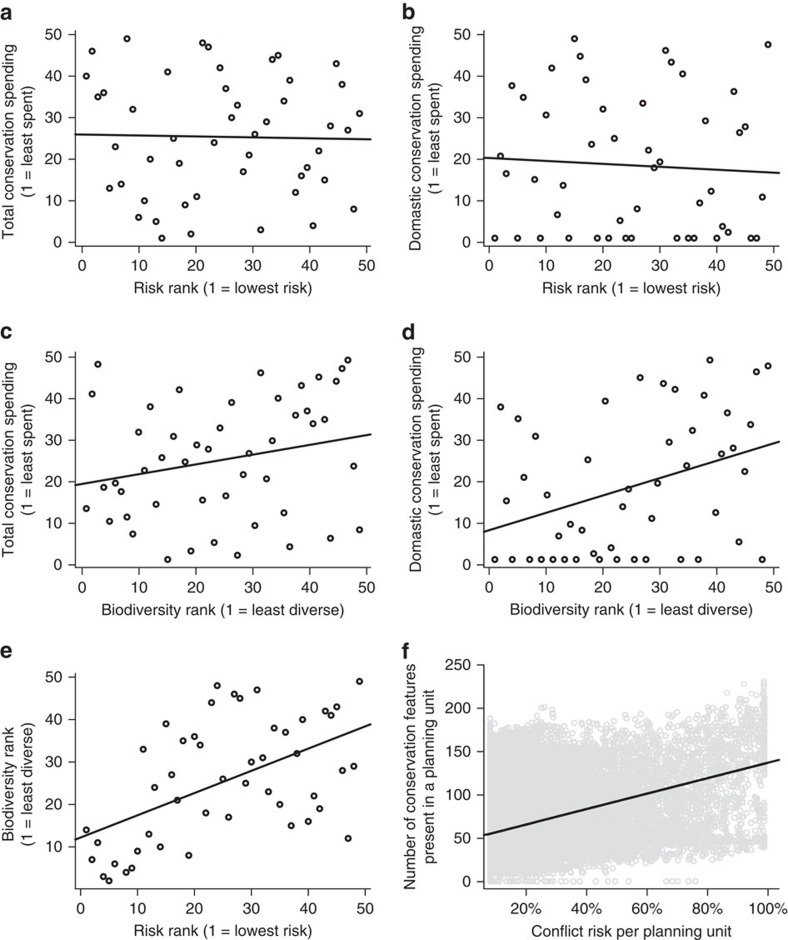
Correlations of risk estimate against conservation spending and biodiversity. We found no correlation between national-level risk and total spending (**a**) or domestic spending (**b**) on conservation. Both total conservation spending (**c**) and domestic conservation spending (**d**) are correlated with national-scale biodiversity (calculated by summing the fraction of each species' range present within a nation). High levels of national-scale biodiversity are correlated with high levels of national-scale conflict risk (**e**). At the scale of the 10-km^2^ planning units, we found a significant correlation between conflict risk and number of conservation features present in a planning unit, with high-conflict-risk planning units tending to contain many endangered mammals (**f**). Conservation spending estimates were acquired from a database compiled by Waldron *et al*.^31^. National-scale estimates of conflict risk were acquired from the Institute for Economics and Peace^26^. Data in **a**–**e** represent ranks, and data in **f** are the values for each planning unit and are shown in grey so the trend line is visible.

We found no correlation between national-scale conflict risk^26^ and international conservation aid^31^ (Spearman's rank correlation, *r*_s_=−0.025, *P*=0.86, *n*=48; Fig. 1a) or between conflict risk and domestic spending on conservation^31^ (*r*_s_=−0.052, *P*=0.73, *n*=48; Fig. 1b). However, we found a significant relationship between biodiversity^31^ and international conservation aid^31^ (*r*_s_=0.30, *P*=0.038, *n*=48; Fig. 1c), and between biodiversity and domestic conservation spending (*r*_s_=0.41, *P*<0.01, *n*=48; Fig. 1d). We also found significant correlations between biodiversity and conflict risk when using national-scale risk estimates^26^ (*r*_s_=0.52, *P*<0.001, *n*=48; Fig. 1e) and fine-scale estimates (*t*=72.29, *P*<0.001, *n*=48; Fig. 1f).

### National-scale versus fine-scale risk estimates

**Figure 2:**
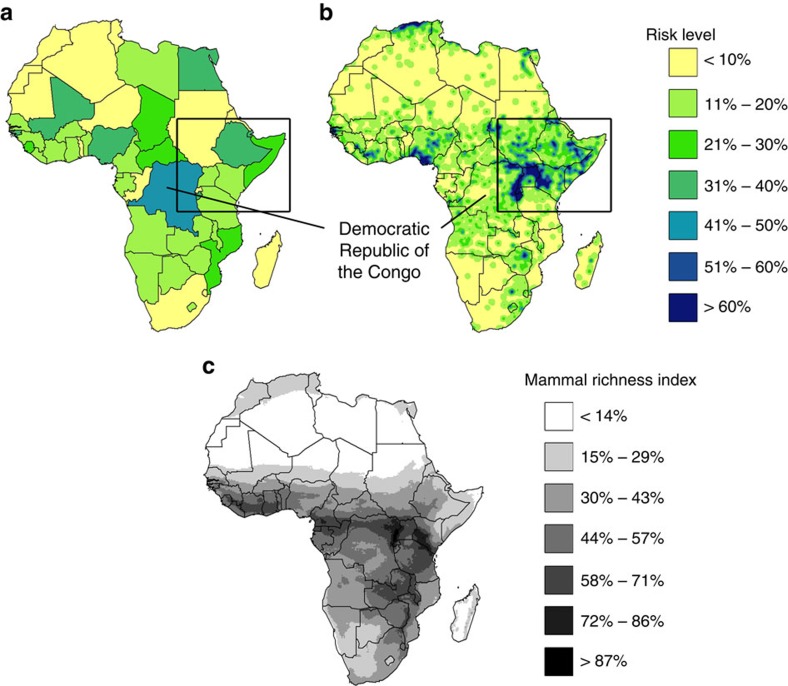
Continental scale risk and biodiversity patterns. (**a**) Risk of conflict from national-scale estimates^26^. (**b**) Fine-scale conflict-risk layer derived from the geographical locations of previous conflicts and the severity of the incident^30^, with the inset identifying the horn of Africa to the Congo Basin displayed in Fig. 3, one of the most conflict-prone but biodiverse regions of Africa (**c**) Mammal species richness index measured as the proportion of 236 threatened mammals that have part of their range in each planning unit.

**Figure 3:**
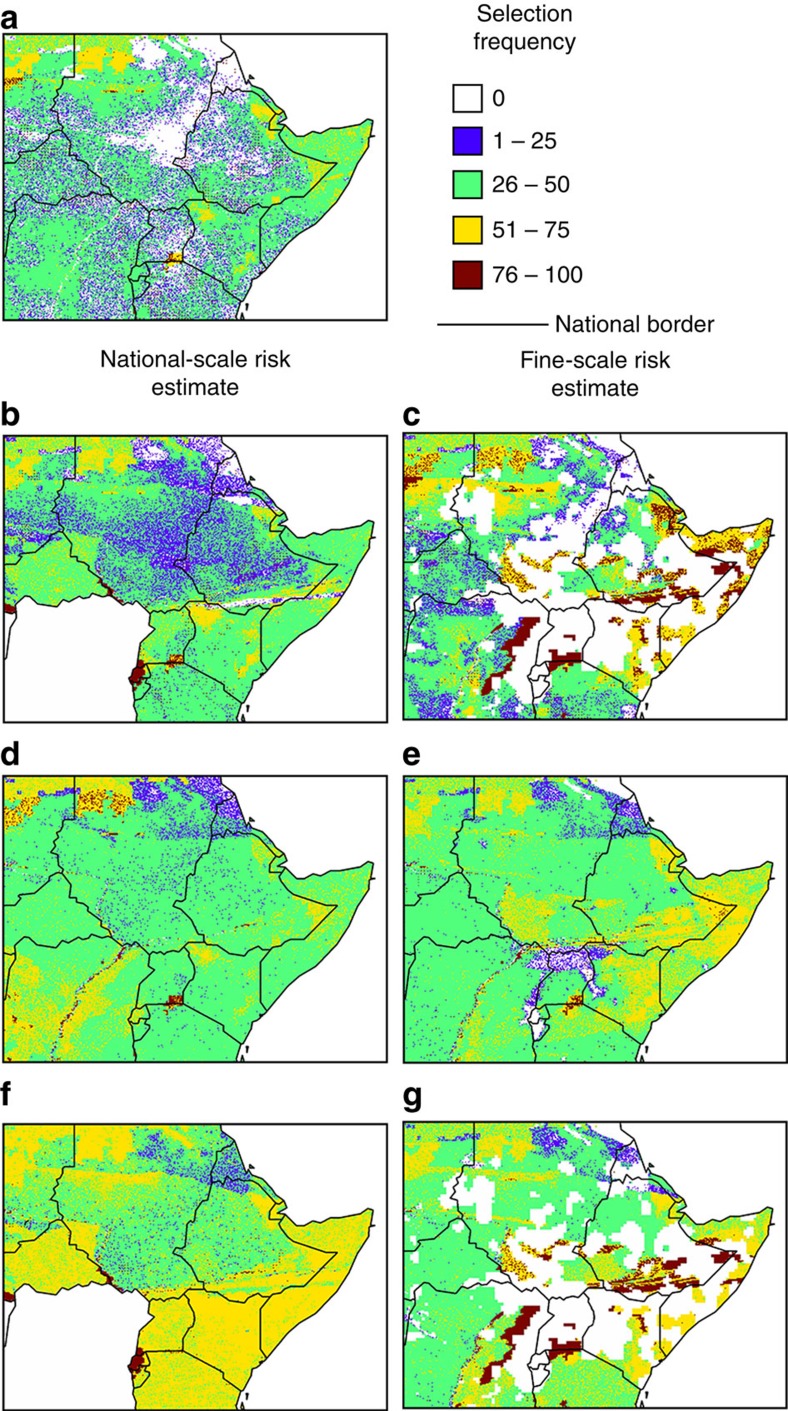
Selection frequencies of planning units under different risk strategies. Panels show the most conflict-prone region of the African continent, including the eastern border of the DRC and the Horn of Africa. The selection frequency of planning units based on biodiversity and cost data alone (**a**) (conflict ignorant). The selection frequency of planning units when areas with >35% risk of conflict are avoided (conflict avoiding) using national-scale (**b**) and fine-scale (**c**) risk data. The selection frequency of planning units when risk was explicitly incorporated (conflict accounting) using national-scale (**d**) and fine-scale (**e**) risk data. The selection frequency of planning units when areas with >35% risk of conflict were avoided, and risk was accounted for in the remaining areas (conflict sensitive) using national-scale (**f**) and fine-scale (**g**) risk data.

**Figure 4:**
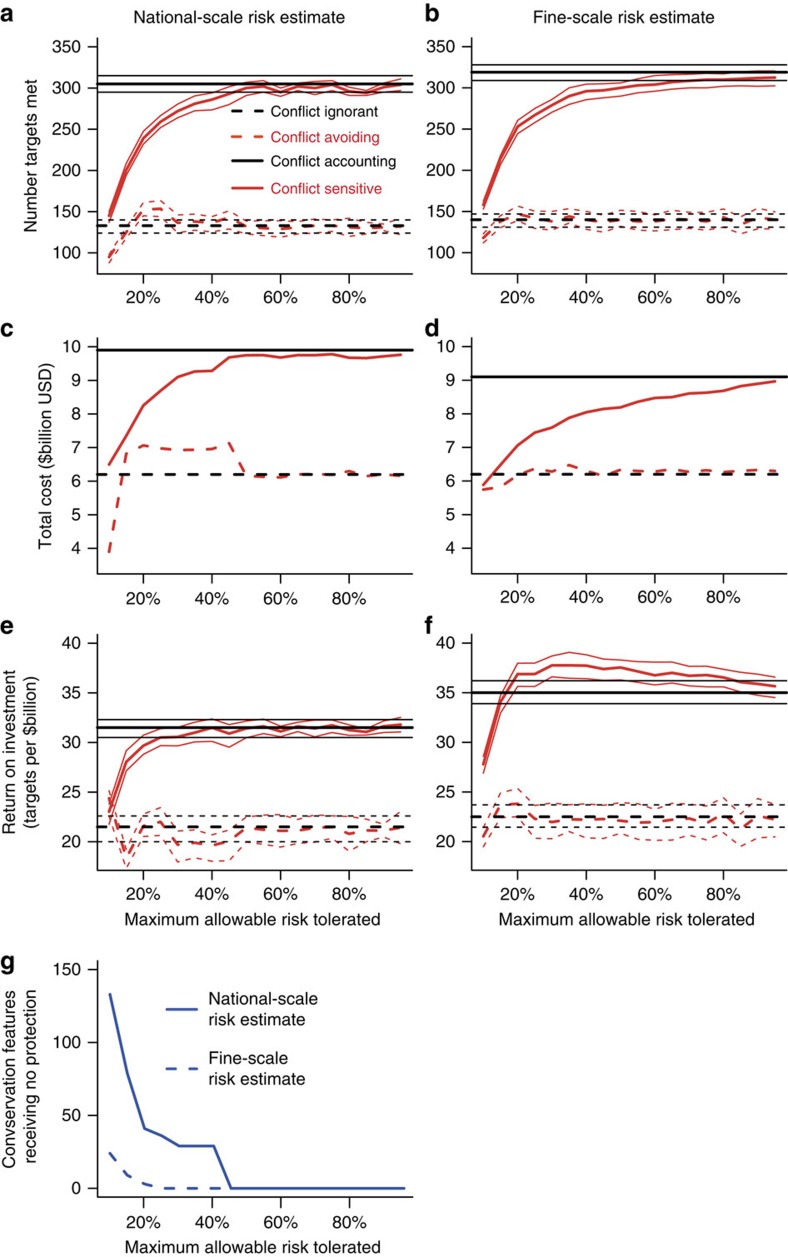
Financial and biodiversity consequences of different attitudes towards risk. The *x* axis relates only to conflict-avoiding and conflict-sensitive strategies. Different lines represent different attitudes. Conflict ignorant represents the outcomes of basing conservation decisions only on biodiversity and cost data. Conflict avoiding represents the consequences of avoiding areas with greater than a maximum allowable risk. Under conflict accounting, the risk of conflict is incorporated into planning decisions along with biodiversity and cost data. A conflict-sensitive strategy represents a combination of the previous two attitudes to risk, areas with a conflict risk greater than a maximum tolerable level are avoided, and risk of conflict is accounted for in the remaining planning units. (**a**,**b**) Number of conservation targets met. (**c**,**d**) Total cost of the protected network in billions of USD. (**e**,**f**) Overall return on investment in terms of conservation targets met per billion USD. (**g**) Number of targets predicted to receive no protection under the two strategies where upper limits are set on maximal tolerable risk (conflict avoiding and conflict sensitive). Data show mean estimates and 95% confidence intervals in all panels except **c**,**d** and **g**, where no error estimates were produced as the data represent the output from the ‘best' protected area network, and are not affected by probabilistic losses due to conflict.

Fine-scale estimates of conflict risk indicated substantial within-country variation (Fig. 2a,b), and illustrated the correlation between conflict risk and biodiversity (Fig. 2a,c). The use of fine-scale data increased the spatial precision with which conflict-risk estimates could be incorporated into planning decisions and altered the selection frequency of the planning units (Fig. 3). Importantly, fine-scale estimates prevented the exclusion of entire nations when high-risk areas are avoided (Fig. 3c,g), and have the potential to improve return on investment (Fig. 4e,f).

### Performance of the four strategies for incorporating risk

Under a conflict-ignorant strategy, many selected planning units would be in high-conflict-risk areas, regardless of whether national-scale or fine-scale risk estimates are used (compare Fig. 2a,b with Fig. 3a). Using national-scale estimates of conflict risk, up to one in three selected planning units would have a conflict risk >25%, a value that drops to one in four when fine-scale risk data are used. As a consequence of many selected planning units being in areas of high conflict risk, this strategy would fail to achieve almost half of the conservation targets (<30% of the current distribution protected; Fig. 4c,d). Although the initial upfront cost of the protected area network would be low (Fig. 4c,d), the low number of targets met would lead to a poor return on investment for the conflict-ignorant strategy (Fig. 4e,f).

When a conflict-avoiding strategy is adopted, at low-risk thresholds (<10%), fewer targets are met than under a conflict-ignorant strategy, regardless of whether national-scale or fine-scale conflict-risk data are used (Fig. 4a,b). When national-scale estimates of conflict risk are employed, and maximum tolerable risk is set between 10 and 45%, there are minimal differences compared with the conflict-ignorant strategy in terms of the number of targets met, despite an increase in cost (Fig. 4a,c). Crucially, when national-scale risk estimates are used, a large number of conservation features are predicted to receive zero protection as they occur exclusively in high-conflict-risk nations. For example, even when the national-scale maximum tolerable risk is set at 40%, the entire ranges of at least 31 species are unavailable for selection (Fig. 4g), a value that is greater still when using alternate estimate of risk from Hegre *et al*.^39^ (Supplementary Note 3). Conversely, using fine-scale data under a conflict-avoiding strategy results in all species receiving at least some protection when the maximum tolerable risk is ⩾25% (Fig. 4g). Regardless of whether national-scale or fine-scale estimates of conflict risk are used, the protected area network does not perform better than a conflict-ignorant strategy in terms of cost, targets met or return on investment (Fig. 4c–f).

Adopting a conflict-accounting strategy would meet the majority (∼95%) of conservation targets (Fig. 4a,b), irrespective of whether national-scale or fine-scale estimates of conflict risk are employed. This increase in the number of targets met is achieved through a combination of opting for lower-risk planning units and increasing the number of planning units selected. This also increases costs compared with conflict-ignorant and conflict-avoiding strategies (Fig. 4c,d). However, the return on investment is improved compared with the conflict ignorant and conflict-avoiding strategies (Fig. 4e,f), as the increase in targets met is proportionally greater than the increase in cost. Under a conflict-accounting strategy, the use of fine-scale data would increase the number of targets met and the overall return on investment (Fig. 4e,f).

The highest overall return on investment could be achieved under a conflict-sensitive strategy using fine-scale conflict-risk data (Fig. 4f), when the maximum tolerable risk is between 30 and 55%. This high return on investment occurs because as the maximum tolerable risk increases, the number of targets met increases more rapidly than the overall cost (Fig. 4b,d). In contrast, selecting areas for protection using national-scale risk data could result in some features receiving no protection if the maximum tolerable risk is low, in the same manner as under a conflict-avoiding strategy (Fig. 4g).

### Analysis of uncertainty in conflict-risk estimates

When the upper 95% confidence limit of the fine-scale risk estimate is used to identify areas for protection, the calculated number of targets met and overall return on investment for conflict-accounting and conflict-sensitive strategies are still greater than when the national-scale estimates of risk are employed (Supplementary Note 2 and Supplementary Figs 3 and 4). The protected area network generated using an alternative national-scale estimate of conflict risk^39^ performs poorest overall in terms of number of targets met or return on investment for all four risk strategies (Supplementary Note 3 and Supplementary Figs 5 and 6).

## Discussion

Historical conservation spending patterns suggest that high biodiversity nations have been favoured regardless of the conflict risk, indicating a willingness to invest in conflict-prone nations when rewards are high^9^. At the local level, however, managers may face severe repercussions for failed projects, leading to risk-averse behaviour^41^. Examples from the United States would suggest that agencies have a low tolerance towards risk of failure^22^,^42^, instead favouring suboptimal actions with low risk^21^. Our results show that this attitude of avoiding high-risk areas or projects is not optimal as a conflict-avoiding strategy performed poorly in terms of targets met and return on investment. We instead propose that managers should be accepting of risk, and account for that risk, to maximize conservation outcomes.

Our spatial prioritization analysis seeks to identify areas that would protect 30% of each conservation feature's range for the minimum overall cost (a ‘minimum set' problem^19^). When conflict risk is not factored into the selection process, there is no motivation to incorporate planning units over the 30% required to meet the target. For many features, the loss of a small number of planning units may be sufficient for the feature to have <30% of its current range protected, meaning that the target is missed. Accordingly, being conflict ignorant is the poorest performing strategy, in terms of number of targets met and overall return on investment.

Choosing to avoid conflict-prone areas would lead to the majority of conservation features not receiving adequate protection. The negative effects of areas being excluded are exaggerated when national-scale estimates of conflict risk are used. When species are endemic to a single, conflict-prone nation, excluding that nation from a protected area network would lead to these species receiving zero protection. For example, if maximum tolerable risk was set at <40%, several iconic conservation features, including the eastern lowland gorilla (*Gorilla beringei graueri*), would be essentially abandoned.

Adopting a conflict-accounting strategy would lead to a ∼50% increase in the cost of establishing the protected area network compared with a conflict-ignorant strategy. However, the ∼50% cost increase generates a protected area network where almost all of the conservation targets are met (a ∼100% increase), increasing return on investment. The conflict-accounting strategy may increase overall conservation success through the following two mechanisms: (1) high-conflict-risk areas are avoided where possible; and (2) additional areas containing conservation features are protected to offset losses predicted to be incurred if conflicts occur^32^,^43^. However, many conflicts occur in close proximity to areas of high population density^44^, meaning that sufficient additional areas may be unavailable. At the local scale, managers may be aware of areas that are unavailable for protection. In a future investigation, deeming these planning units unavailable would force the software to include planning units elsewhere^19^.

When national-scale conflict risk estimates are used, a strategy of avoiding the most conflict-prone regions and accounting for risk in the remainder (conflict-sensitive) yields a similar return on investment to conflict-accounting. Conversely, when fine-scale risk estimates are used, between risk thresholds of 30% and 55%, a conflict-sensitive strategy yields a greater return on investment than conflict-accounting. Although a conflict-sensitive strategy yields the highest return on investment, it also leads to the exclusion of high-risk areas. This exclusion of high-risk areas may be appealing to conservation managers wishing to limit the exposure of staff to conflict, but means any conservation feature existing predominantly in high-risk areas may be abandoned when maximum tolerable risk is low. The decision to opt for a conflict-accounting versus a conflict-sensitive strategy therefore depends on whether the goal is to achieve the highest return on investment or to save the most species, and be influenced by the risk tolerance of conservation investors and managers^22^.

For the two strategies that account for risk (conflict-accounting and conflict sensitive), the fine-scale risk estimate is predicted to outperform national-scale estimates in terms of targets met, cost and returns on investment (even when the upper 95% confidence limit of the risk estimate is used; Supplementary Note 2 and Supplementary Fig. 4). National-scale estimations of conflict risk incorporate many parameters including institutional consistency and economic openness^45^. However, incorporating national-scale calculations into systematic conservation planning assumes that conflict risk is homogenous within a nation, an assumption that is likely to be false. For example, the DRC is historically among the most unstable nations in Africa^26^,^45^, but conflict risk within the nation is highly heterogeneous. The majority of conflicts occur around the eastern border^30^ (Fig. 2b), leaving a relatively safer area to the west^30^. The DRC also contains some of Africa's highest numbers of mammalian species (Fig. 2c)^46^. High-priority conservation areas in countries such as the DRC could be excluded from a future protected area network if investment decisions were based on national-scale risk data, potentially incurring consequences for both economic development and the achievement of international biodiversity targets.

The results we present do not include the social impacts of conflict, either the direct impact on local people or the potential loss of ecosystem services^47^. Conflict can reduce a society's access to potable water^48^ and damage soils^49^, potentially increasing pressure on remaining ecosystem services. The inclusion of ecosystem service targets, when planning for protected areas, may therefore be important in conflict regions, where resources are likely to be scarce^50^.

Our measures of conflict risk do not explicitly estimate the impact of conflict on conservation features; however, information on the differential vulnerability of all species to the impacts of conflict could be included if this information is developed for lesser known species^22^,^51^. We have therefore assumed that should a protected area be impacted by a conflict, it will lead to the loss of all conservation features that inhabit that area. This pessimistic, ‘worst case' assumption increases the chances of conservation targets being met when information is imperfect.

We acknowledge that the dynamic nature of conflicts means they may have disparate effects on conservation^7^,^17^. For example, rebel activity may require locally sourced natural resources to fund conflict activities^52^, thereby placing a higher demand on the local environment than an externally funded inter-state war. Our results will be sensitive to the differing impacts of various conflicts; however, the sensitivity analysis we present (Supplementary Note 2) demonstrates that when the conflict-risk estimates are increased or decreased (analogous to differences in impact), the pattern of performance for the four risk strategies remains the same. We therefore suggest that although the quantitative outcome of a conflict-accounting and conflict-sensitive risk strategies will be sensitive to the impact of different conflicts, both strategies would still outperform conflict-ignorant and conflict-avoiding strategies.

Our analyses utilized estimates from multiple sources that employed different techniques to estimate conflict risk. In the main text, we used the ‘Peace Score', a national-scale estimate produced by the Institute for Economics and Peace^26^. The Peace Score is generated using a five-point scale (determined by expert opinion) for 23 different indicators, divided into three domains (ongoing conflict, national harmony or discord and national militarization). The Peace Score therefore uses a substantial volume of information, likely making it a robust metric. The second national-scale estimate of conflict risk extracted from Hegre *et al*.^39^ (Supplementary Note 3) uses a national-scale logistic model, with predictors such as population size, infant mortality, ethnic antagonism and neighbourhood characteristics^39^. While these national-scale estimates of conflict risk may be robust owing to the volume of information used, their lack of spatial precision leads to difficulties in planning at the sub-national level. Conversely, our fine-scale estimate of risk used far fewer metrics (history of local incidents, severity of local incidents and time since last local incident), but demonstrates the value of fine-scale data in a similar manner to increased precision of biodiversity and cost data^19^,^24^.

Our results demonstrate the value of incorporating the risk of armed conflict into systematic conservation planning and illustrate the importance of local-scale data. We hope that protected area managers solicit estimates of the potential conflict risks at the local scale, and incorporate this information using the principles we have demonstrated to maximize conservation outcomes. We would hope our results increase the confidence of managers, governments and the public to accept some level of risk while undertaking conservation actions. This willingness to accept risk will allow conservation decisions to be based on likely outcomes, rather than a potentially suboptimal fear of failure^21^.

## Methods

### Data on historical patterns of conservation spending

We obtained species distribution maps from previously published sources^31^,^53^, then calculated two metrics for biodiversity. The first national-scale metric summed the fractions of each mammalian species' range that exist within each nation^31^ (Fig. 1a–e). The second, local-scale metric quantified the number of endangered mammals that had part of their range within a planning unit (Fig. 1f)^53^.

### Identification of future protected areas

We identified priority areas for future conservation investment under the four different risk strategies using spatial prioritization^19^. A 10-km^2^ grid was superimposed over Africa, producing 335,694 planning units available for investment. We identified 357 conservation features comprising Africa's 121 ecoregions^54^, and the ranges of 236 threatened African mammal species (same data as used to calculate local-scale metric for biodiversity in Fig. 1f (ref. 53)). Our conservation goal was to represent 30% of each feature in the protected area network, representing the recommended minimum for terrestrial ecosystems^40^. Risk of conflict was incorporated using two different methods. We obtained published estimates of conflict risk at the national scale^26^,^39^, and also calculated risk at the resolution of the 10-km^2^ grid using historical conflict data^30^. As locations with a history of conflict are predicted to have a higher future risk^26^, we produced a logistic model of predicted risk within a planning unit based on local conflict history. Since the effect of conflicts can be felt in surrounding areas through movement of conflict actors or refugees^11^, we created 30 km ‘impact zones' around incidents, and planning units within the impact zone were deemed to have experienced the conflict. We used the conservation planning software Marxan^55^ to identify areas for conservation under each different risk strategy. Below we go through each of the steps necessary to perform the analysis in term.

### Calculating estimated land costs

Each planning unit was assigned a financial cost (USD) value that included the following three core components: the cost of purchasing the land, the foregone agricultural revenue over 5 years and the cost of protected area management^53^.

### Spatial data for conservation targets used in spatial prioritization

Spatial data were obtained describing the distributions of Africa's 236 terrestrial mammal species classified as >threatened or data-deficient according to the IUCN red list of endangered species^56^, and Africa's 121 unique ecoregions (data are available at https://disturbance.s3.amazonaws.com/EcoregionalRollup.zip). To determine the biodiversity value of each planning unit, we calculated the proportion of the total area of each conservation feature represented in each planning unit. When a planning unit was selected to be part of the protected area network, it contributed in meeting the protection target, which is 30% of the current extent of that conservation feature^19^.

### Conflict data and risk calculation

The definition of ‘risk' we employed incorporates the following two dimensions: the chances a conflict incident will occur and the loss or damage associated with the event^22^. Data on conflict risk were obtained through two different methods. First, we sourced national-scale estimates from the scientific literature^26^,^39^, we focused on the more recently compiled data from the Institute for Economics and Peace^26^ as opposed to the data from Hegre *et al*.^39^. Second, we used geo-referenced data on previous conflict incidents^30^ at the sub-national level. The geo-referenced conflict data allowed us to estimate conflict risk at the planning unit scale (10 km^2^), and were obtained from the Armed Conflict Location and Event Dataset (ACLED)^30^. Under the assumption that following an incident people are likely to move away, we assumed that conflict incidents affected the planning units close to where they took place, and included 30 km ‘impact zones' around each conflict incident. This distance was chosen as it is greater than the average distance travelled by paramilitary groups and refugees per day^57^. We opted to use the ACLED database as opposed to the UCDP/PRIO^58^ database owing to its high level of spatial precision and number of data points. Many different definitions of what constitutes a conflict exist, including the Toronto Group's definition of >1,000 battle deaths, with 50% casualties per side, and the conflict classification used by Hegre *et al*.^39^, where incidents with 25–999 battle deaths per year, are considered minor, while >1,000 deaths represent major conflicts^39^. These definitions could, however, overlook many of the small, local-scale conflicts that may be important from a conservation perspective.

We built a statistical model to calculate the planning unit-specific conflict risk using local history of conflicts. To calibrate the model, we used the data from 10 years of the ACLED database (1999–2008) to ‘predict' risk in the subsequent 5 years using the presence/absence of an incident in each planning unit between 2009 and 2014 as a response variable. The model fitted a logistic curve using the following three significant descriptive variables (bounded by the years 1999–2008): the number of previous incidents in that planning unit (probability of future conflict, logistic regression, *z*=37.44, *n*=335,694, *P*<0.001), the number of fatalities (likely impact of conflict, *z=*25.51, *P*<0.001) and years since the last incident (probability of future conflict, logistic regression, *z*=25.54, *n*=335,694, *P*<0.001). The logistic model generated probabilistic outputs (conflict-risk estimates between 1 and 100%) for each planning unit. The model accounted for 35% of the total variation in the data (calculated using McFadden's pseudo-*R*^2^). Full parameter estimates for the logistic model and relationships between the descriptive parameters and conflict risk are given in Supplementary Note 1, Supplementary Table 1 and Supplementary Fig. 2. Following parameterization of the logistic model, we used conflict data from the years 2005 to 2014 to predict the risk of conflict incidents during the next 5 years (2015–2019). We are aware that our logistic model likely represents an oversimplification of the factors that can lead to armed conflicts as well as their likely damage; however, the simplicity of the model allows it to be applied to the entire continent.

### Spatial prioritization

Systematic conservation planning was conducted using the conservation decision-support software Marxan^24^,^55^. For all conservation features, we used the recommended target of 30% of the current extent of the feature^40^. Planning units were designated as ‘existing protected areas' if >50% of the area of a planning unit already fell within a protected area, and these areas were forcibly included in the protected area network. Marxan was run 100 times, and the frequency with which available planning units were selected within the 100 Marxan runs indicated their relative priority (Fig. 3).

### Monte Carlo simulations to estimate return on investment

In order to estimate the return on investment yielded by each of the conflict-risk strategies, we used Monte Carlo simulations to estimate how many conservation targets were predicted to not be achieved. We assigned a random number between 1 and 100 to each planning unit in the protected area network. If the random number assigned to the planning unit was less than its estimated conflict-risk percentage, that planning unit was deemed ‘lost'. For each conservation feature, the total area of its distribution present in the remaining planning units (not ‘lost') was calculated and compared with the conservation target. If 30% or more of the conservation feature's original area was contained in the remaining planning units, the conservation target was deemed met. For the best protected area network selected under each risk strategy (out of the 100 Marxan runs), we repeated this procedure 1,000 times. The 1,000 simulations enabled us to produce a distribution of the estimated total number of targets met under each attitude towards risk, allowing the reporting of medians and confidence limits. To estimate return on investment, the predicted performance of the protected area network (number of conservation targets met) was divided by the cost of the protected area.

### Sensitivity analysis

Because our fine-scale estimate of conflict risk has an error estimate associated with it, we re-ran the analyses using the upper and lower confidence intervals. Continental-scale risk estimates using the upper and lower confidence limits, and the results of using these estimates in terms of number of conservation targets met, cost and return on investment are given in Supplementary Note 2 and Supplementary Figs 3 and 4.

The national-level estimates of conflict risk we obtained from published sources did not contain an error calculation^26^. We therefore re-ran the analysis (identified areas for protection under the four different risk strategies and used Monte Carlo simulations to quantify the success of these protected areas) using another set of data from another online source^39^. Full results are given in the Supplementary Note 3 and Supplementary Figs 5 and 6.

## Additional information

**How to cite this article:** Hammill, E. *et al*. Factoring attitudes towards armed conflict risk into selection of protected areas for conservation. *Nat. Commun.* 7:11042 doi: 10.1038/ncomms11042 (2016).

## Supplementary Material

Supplementary Information Supplementary Figures 1-6, Supplementary Table 1, Supplementary Notes 1-3 and Supplementary References.
