# Supplementary material for: Factoring attitudes towards armed conflict risk into selection of protected areas for conservation
Source: Nat Commun. 2016 Mar 30;7:11042. doi: 10.1038/ncomms11042 (PMC4820849; doi:10.1038/ncomms11042)
Supplement: Supplementary Information — Supplementary Figures 1-6, Supplementary Table 1, Supplementary Notes 1-3 and Supplementary References. [file ncomms11042-s1.pdf]

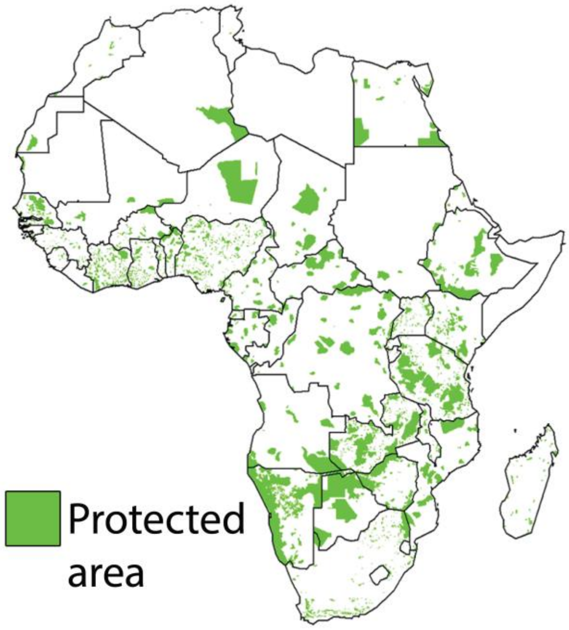

Supplementary Figure 1. Extent of current protected area network.

Africa currently has approximately 2.2 million km^2 (7%) of its landmass in protected areas, this is considerably less than the global average of 13%.

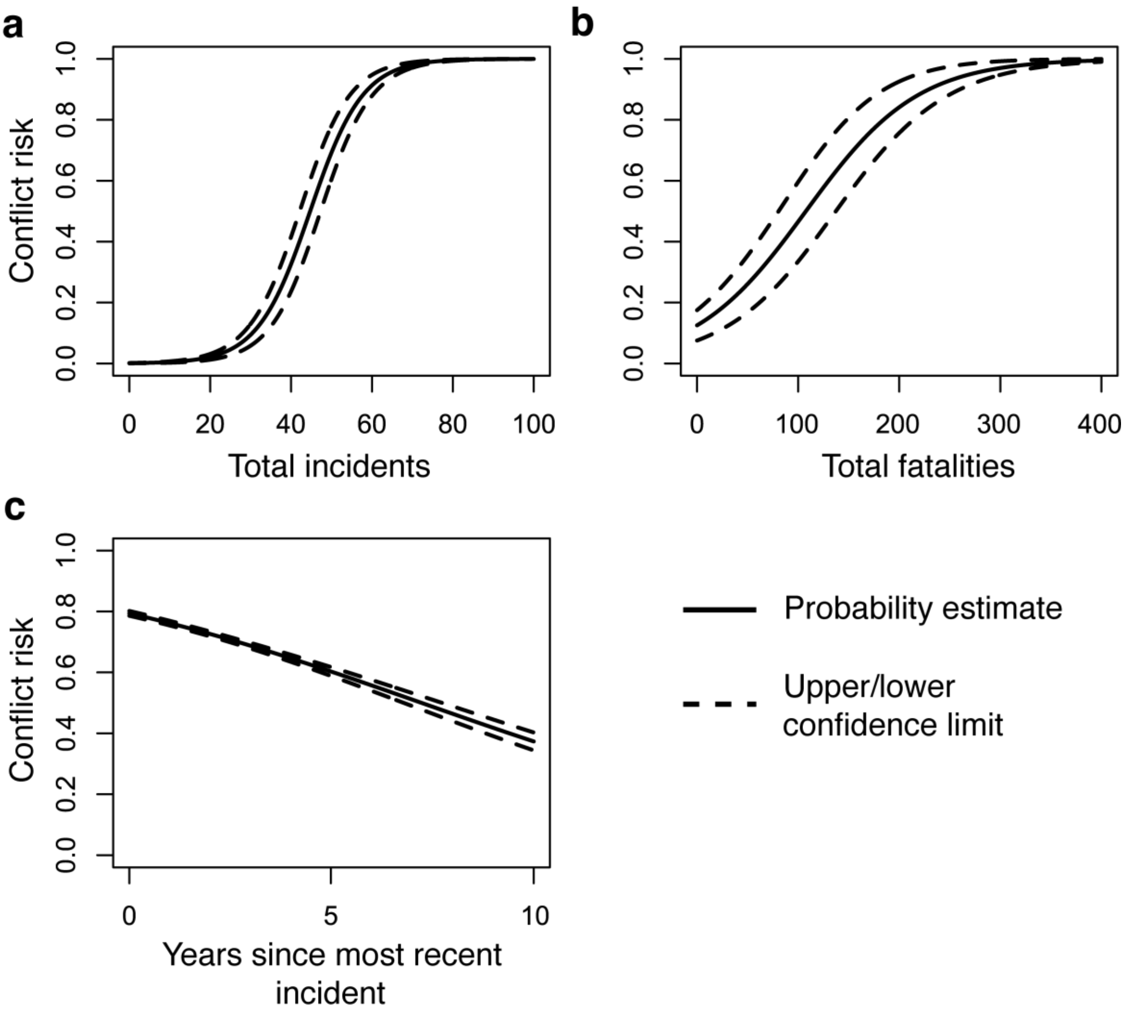

Supplementary Figure 2. Modeled data showing the relationships between the predictor variables and conflict risk for the years 2009-2014. **a.** Conflict risk is greater in planning units that have experienced a larger number of conflicts historically. **b.** A greater number of fatalities are associated with higher levels of conflict risk. **c.** Conflict risk declines with a greater number of years since the most recent conflict incident. For all panels, the values of the two predictor variables not being illustrated were set at their mean value.

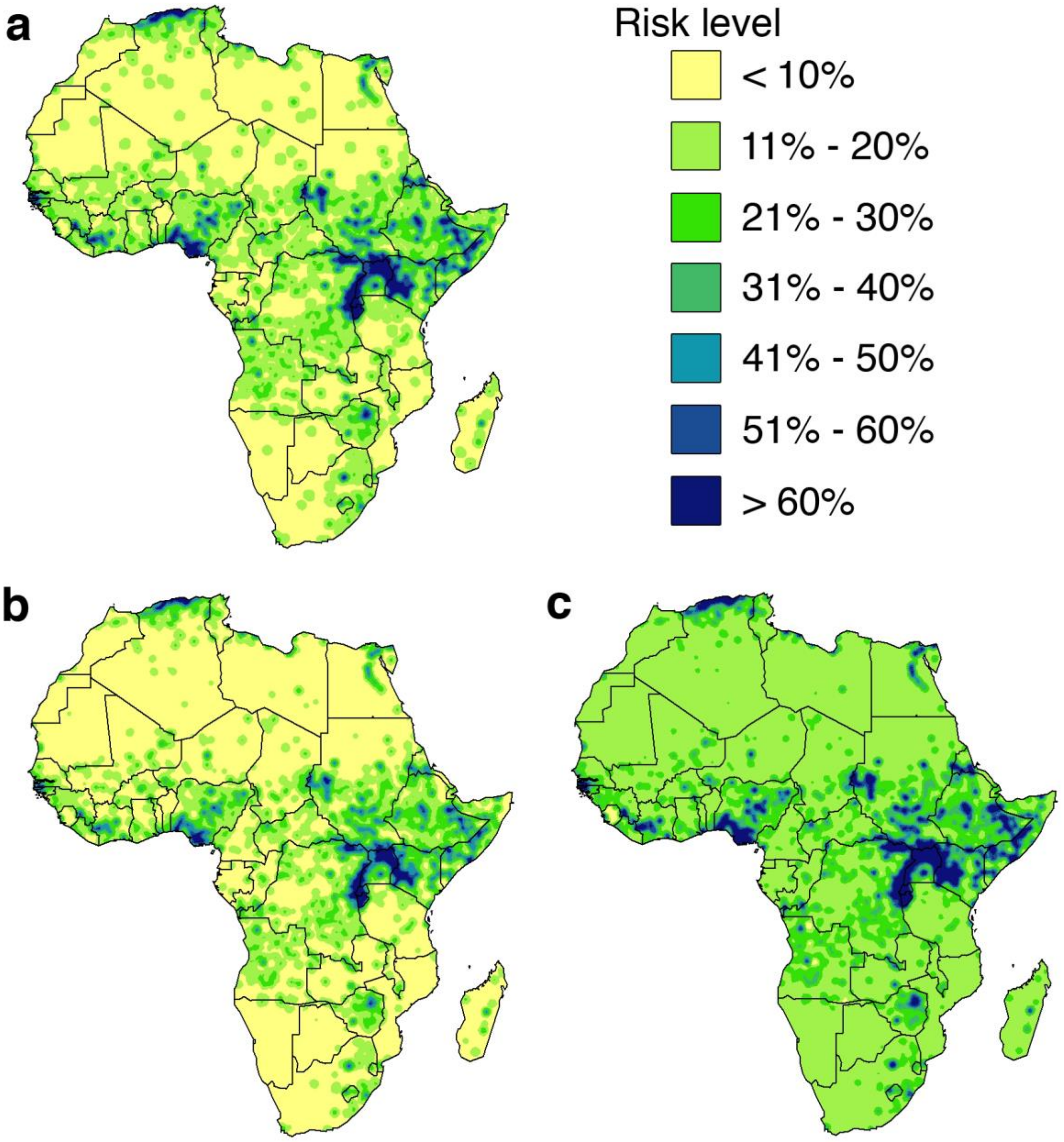

Supplementary Figure 3. Fine-scale estimates of conflict risk generated from the logistic model. **a.** Conflict risk estimates generated from parameters estimates given in Table S1. **b.** Risk estimates generated using the lower confidence limit from the logistic model. **c.** Risk estimates generated using the upper confidence limit from the logistic model.

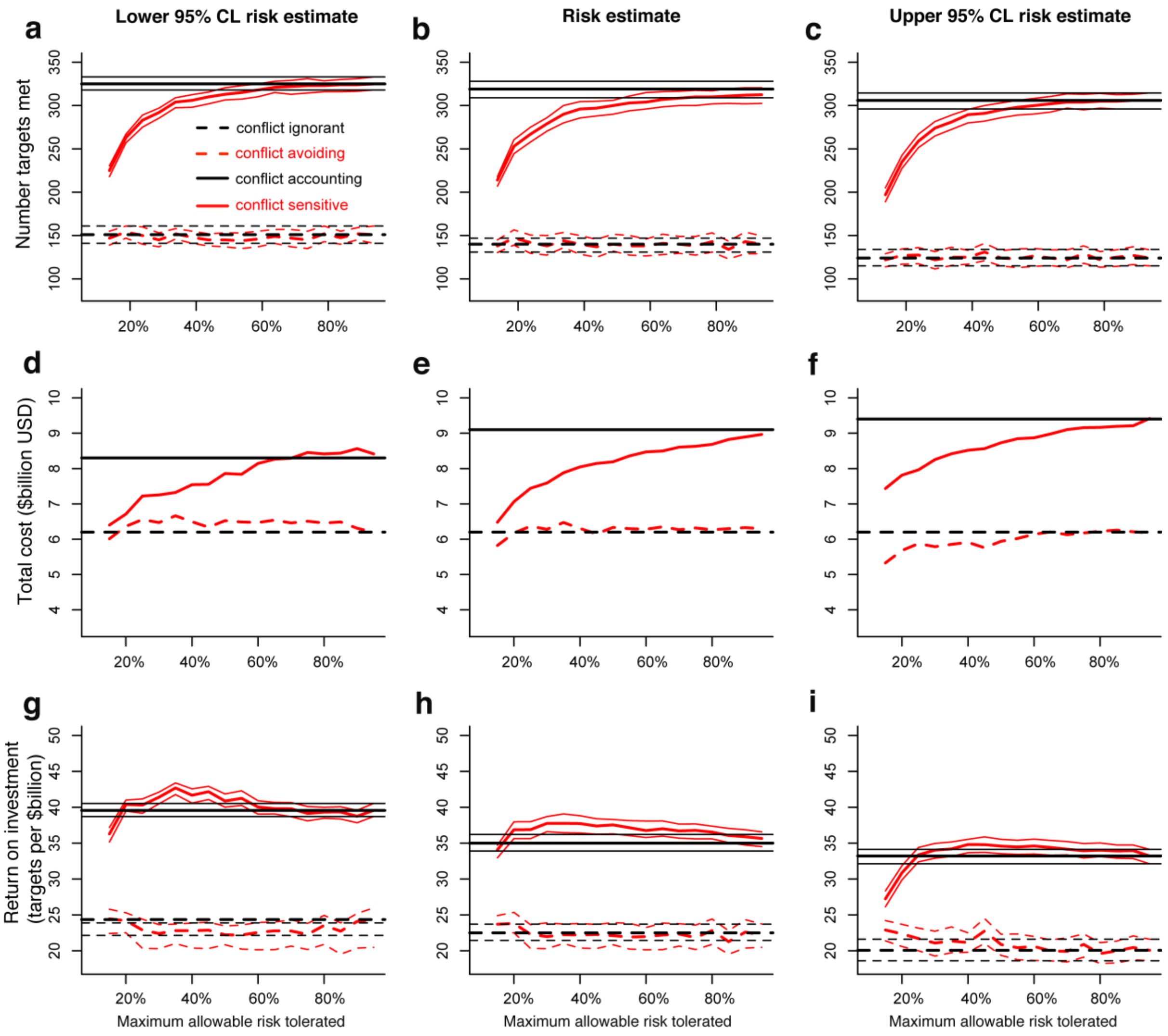

Supplementary Figure 4. Financial and biodiversity consequences of using the upper and lower 95% confidence limits of the fine scale-estimate of conflict risk. (**a-c**) number of conservation targets met, (**d-f**) cost of the protected area network, (**g-i**) overall return on investment.

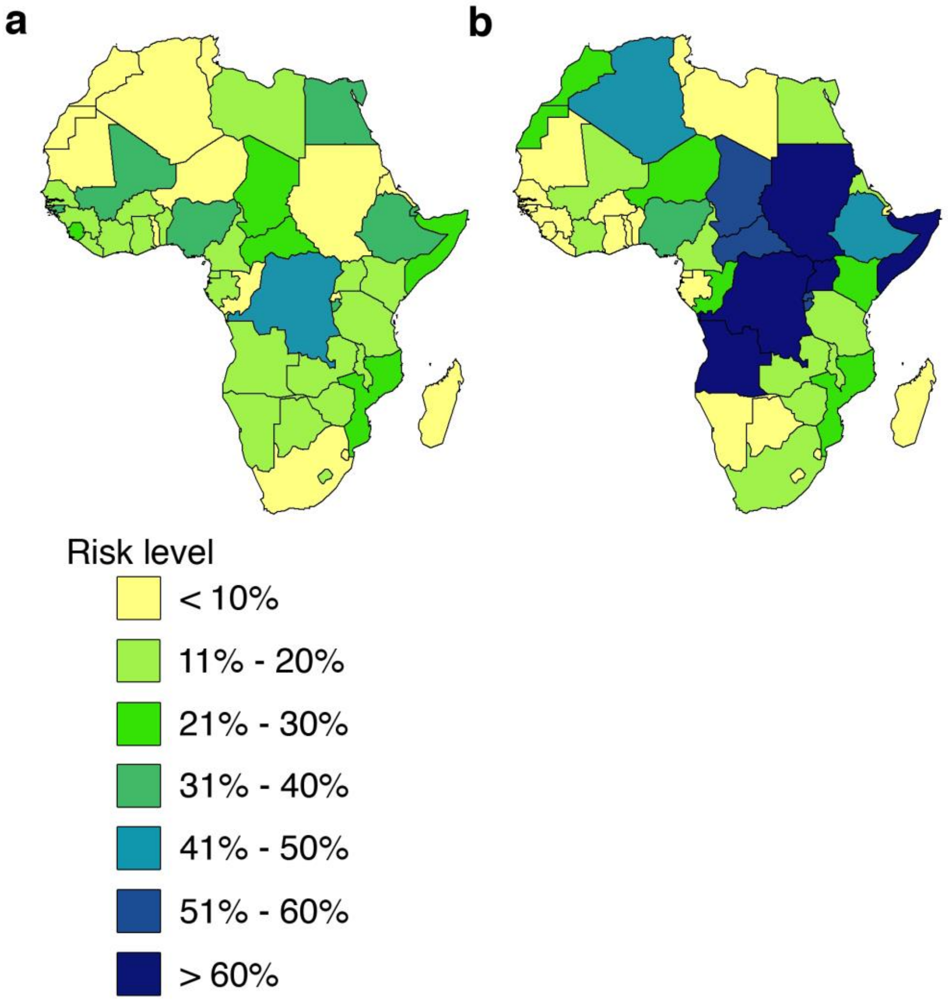

Supplementary Figure 5. National-scale estimates of conflict risk for the years 2015-2019 obtained from (**a)** the Institute for Economics and Peace[3], and (**b**) Hegre *et al* (2013)[2].

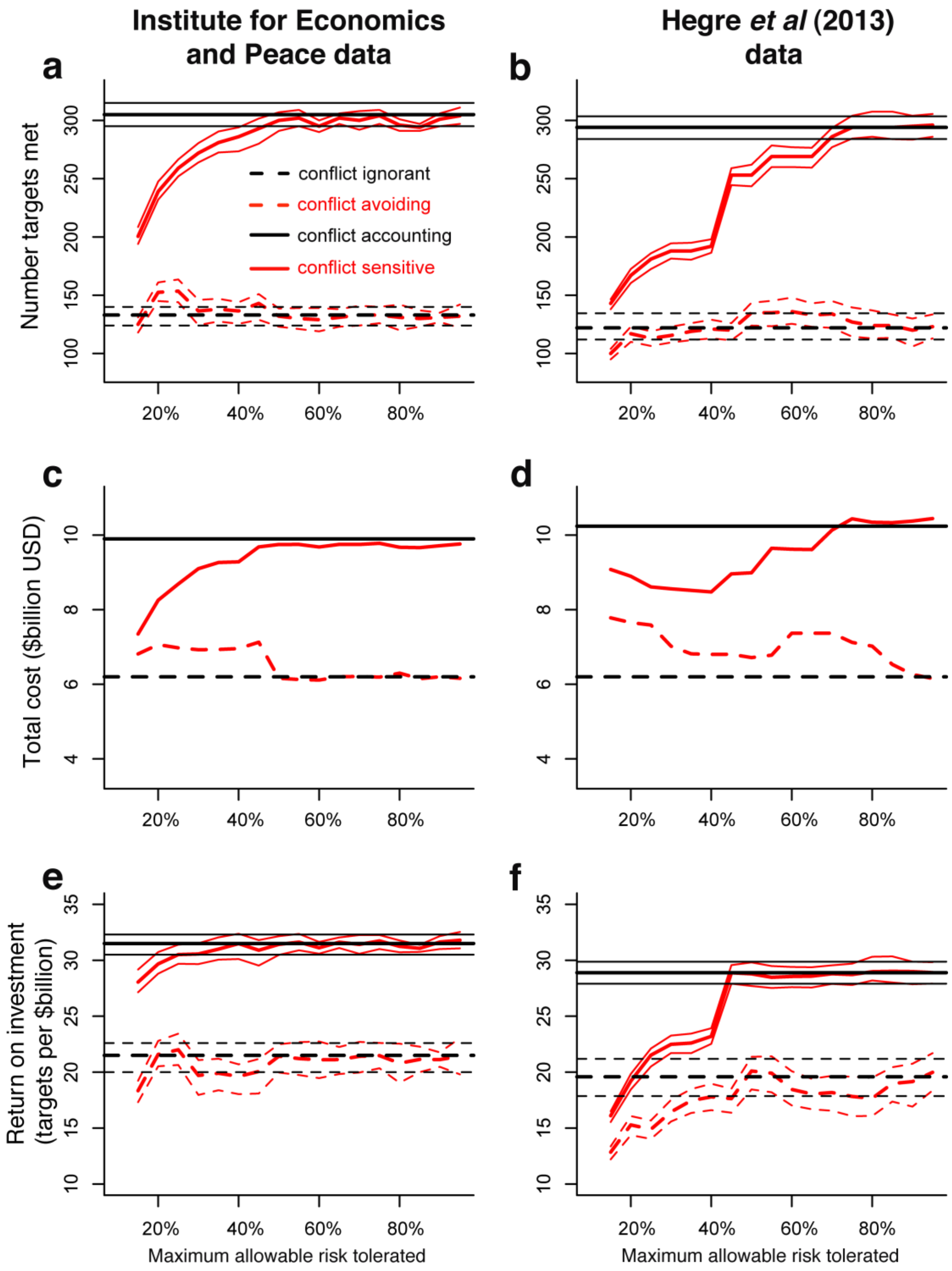

Supplementary Figure 6. Performance of the protected area networks generated using data from the Institute for Economics and Peace, and Hegre *et al* 2010 under the four different strategies to dealing with conflict risk. (**a-b**) number of conservation targets met, (**c-d**) cost of the protected area network, (**e-f**) overall return on investment.

Parameter	Estimate	St Error	z	P
**Fatalities**	0.0002	0.0000085	21.25	<0.001
**Years since**	0.12	0.0049	25.54	<0.001
**Total incidents**	0.1500	0.0041	37.44	<0.001

Supplementary Table 1. Parameter estimates for the logistic model predicting future conflict risk at a fine-scale.

Supplementary Note 1 - Generation of the fine-scale risk estimate

Logistic model used to generate the fine-scale risk estimate.

We used the glm() function in R[1] to produce a logistic model predicting conflict risk in each planning unit based on that planning unit's conflict history. The conflict risk estimate is a combination of two elements, the probability of a conflict occurring, and the impact of that conflict. The model used the total number of conflict incidents, the number of fatalities and the years since the last conflict. Parameter estimates are given in Supplementary Table 1 and the relationship between each predictor variable and conflict risk is shown in Supplementary Fig. 2. Overall, the model accounted for 34.6% of the total variation in the data.

Supplementary Note 2 - Sensitivity of the performance of the four different risk strategies to the fine-scale conflict risk estimate.

The composition and performance of the protected area networks generated by the different risk strategies will depend on the precision and accuracy of the fine-scale risk estimates. Here we investigate how uncertainty in the fine-scale risk estimate affects the overall conclusions by repeating the analyses using the upper and lower 95% confidence limits on the fine-scale risk estimate.

Supplementary Fig. 3 shows how the use of the upper and lower confidence limits alters the fine-scale risk estimate at the continental scale. The original risk estimate is illustrated in Supplementary Fig. 3a. When the lower confidence limit is used, the extent of the highest-risk areas are reduced (Supplementary Fig. 3b). However, when the upper confidence limit of conflict risk is used very few planning units have conflict risk estimates of < 10%, and the extent of the highest risk areas is increased, particularly in the eastern Democratic Republic of the Congo, the Niger delta, and the Horn of Africa (Supplementary Fig. 3c).

For all four risk strategies, using the lower confidence limit conflict risk data leads to an increase in the number of targets met (Supplementary Fig. 4a), a decrease in overall cost (Supplementary Fig. 4d) and an increase in overall return on investment (Supplementary Fig. 4g). Conversely, the use of the upper confidence limit from the conflict risk estimate reduced the number of targets met (Supplementary Fig. 4c), increased the cost (Supplementary Fig. 4f), and reduced the overall return on investment (Supplementary Fig. 4i). However, the general pattern of performance for the four different risk strategies remained the same, with

“conflict ignorant” and “conflict avoiding” being out performed by “conflict accounting” and “conflict sensitive”.

Supplementary Note 3 - An alternative estimate of national-scale conflict risk.

The national-scale estimate of conflict risk obtained from the Institute for Economics and Peace (IEP) did not include any calculation of error. In order to assess the sensitivity of our results to the national-scale estimates of conflict risk that we employ, we reran the analysis with an alternative estimate of conflict risk at a national-scale. Hegre *et al* (2013)[2] provides estimates of the probability of minor and major conflicts for the years 2011-2050. We therefore extracted their estimates of the probability of major conflicts (> 1000 battle deaths per year) for each of the African nations. Across the continent, the data from Hegre *et al* (2013) estimated total risk to be 23.9% higher, although 25 of the 54 countries were predicted to have a higher level of risk in the IEP data. National-scale estimates of risk obtained from the two sources are given in Supplementary Fig. 5. However, the data provided by Hegre *et al* (2013) do not incorporate any estimate of the impact of conflict, potentially explaining why the overall estimate is higher.

Numerical simulations show that the relatively higher conflict risk estimates obtained from Hegre *et al* (2013) meant that the protected area network generated using the four different risk strategies would be predicted to not perform as well as those generated from the IEP data (Supplementary Fig. 6): fewer conservation targets would be met (Supplementary Fig. 6a and 6b), the cost would be greater (Supplementary Fig. 6c and 6d), and the overall return on investment would be reduced (Supplementary Fig. 6e and 6f). The qualitative pattern of the performance of the different conflict risk strategies remained the same, with “conflict ignorant” and “conflict avoiding” out-performed by the “conflict accounting” and “conflict sensitive” strategies. We opted to use the estimate from the Institute for Economics and Peace, rather than Hegre *et al* (2013), in the main analysis as the former was conducted more recently (2014 as opposed to 2009).

Supplementary References

1. R Core Team. A language and environment for statistical computing. (eds). R Foundation for Statistical Computing (2014).

2. Hegre H, Karlsen J, Nygård HM, Strand H, Urdal H. Predicting Armed Conflict, 2010-2050. *International Studies Quarterly* **57**, 250-270 (2013).

3. Institute for Economics and Peace. Global Peace Index 2013. (eds) (2013).
